# Editorial: Recent Developments in Haploidentical Hematopoietic Cell Transplantation: Therapy and Complications

**DOI:** 10.3389/fimmu.2021.746221

**Published:** 2021-08-17

**Authors:** Ying-Jun Chang, Qing Ding, William Ying Khee Hwang, Ranjit Kumar Sahoo

**Affiliations:** ^1^Peking University People’s Hospital & Peking University Institute of Hematology, National Clinical Research Center for Hematologic Disease, Beijing Key Laboratory of Hematopoietic Stem Cell Transplantation, Beijing, China; ^2^Thomas E. Starzl Transplantation Institute, University of Pittsburgh, Pittsburgh, PA, United States; ^3^Division of Medical Oncology, National Cancer Centre Singapore, Singapore, Singapore; ^4^Department of Medical Oncology, All India Institute of Medical Sciences, New Delhi, India

**Keywords:** stem cell transplantation, graft-*versus*-host disease, graft failure, graft-*versus*-leukemia effect, car-t, infection

The successful application of haploidentical hematopoietic stem cell transplantation (haplo-HSCT) worldwide has made it a reality that almost every allograft candidate has a donor. In the past two decades, significant advances had been achieved in the field of haplo-HSCT. Currently, the outcomes of haplo-HSCT are not inferior to those of other transplant modalities, including human leukocyte antigen (HLA)-matched sibling donor transplantation (MSDT), umbilical cord blood transplantation, and HLA-identical unrelated donor transplantation. Impressively, the numbers of haplo-HSCT increased rapidly in Asia, Europe, and United States of America in the past ten years, especially in China, where the cases of haploidentical allograft exceeded MSDT since 2013. However, complications after transplantation, such as graft failure (GF), leukemia relapse, and graft-versus-host disease (GVHD) are the main bottlenecks for further improving outcomes of haplo-HSCT. Therefore, there is an urgent need to understand the underlying mechanisms and to establish novel strategies for the prevention and treatment of the abovementioned complications in order to improve haploidentical allograft outcomes.

## Immune Tolerance

The successful clinical application of haplo-HSCT is determined by the donor and host T-cell alloreactivities, which lead to unacceptably high incidences of GF and GVHD. Strategies for crossing HLA barriers in the haplo-HSCT modalities include immune tolerance induced by either granulocyte-colony-stimulating factor primed grafts and antithymocyte globulin (ATG) or post-transplant cyclophosphamide as well as *ex vivo* T cell depletion. Further elucidating the underlying mechanisms of immune tolerance in the haplo-HSCT settings would contribute to clinical developments with respect to the lower incidence of GF and GVHD. Original research reported by Weber et al. identified the interferon-γ pathway as the target for exploring therapeutic strategies against GF especially for patients who underwent haplo-HSCT. In the two review papers, Yang et al. summarized recent advances on T cell tolerance, discussing how regulatory T cells maintain self-tolerance either in early life or in allogeneic transplant settings. Hong et al. focused on the roles of antigen presenting cells (APCs), such as dendritic cells, macrophages, played in the pathophysiology of chronic GVHD. They discussed potential new therapeutic approaches targeting APCs for chronic GVHD. Overall, these primary and review papers delineate the mechanisms of GF, T cell tolerance, and chronic GVHD, which provide insights into the treatment for both GF and chronic GVHD.

## Chimeric Antigen Receptor T-Cell

The use of chimeric antigen receptor T-Cell (CAR-T) therapy has changed the landscape for the treatment of relapsed or refractory acute lymphoblastic leukemia. Zhang and Huang not only discussed the complementary anti-leukemia mechanisms on combination of CAR-T cell therapy with allogeneic HSCT, but also provided evidence suggesting the role of CAR-T cell in post-transplant relapse and peri-transplant residual leukemia cell eradication. In addition, CAR technology could be incorporated into the strategy for GVHD treatment. The report from a multi-center retrospective study by Yan et al. demonstrated different characteristics and risk factors of cytokine release syndrome in different B-cell hematological malignancies, suggesting which should be treated individually. Both the aforementioned strategies could further improve transplant outcomes of patients with lymphoblastic malignancies.

## Leukemia Relapse and Virus Infection

For patients who underwent allogeneic HSCT, particularly haplo-HSCT, relapse remain the main cause of death. Furthermore, viral infections is also an important cause of morbidity and mortality in those patients. Zhao et al. reported the association of decreased inhibitory killer immunoglobin-like receptor (iKIR) HLA C with transplant outcomes of patients with myeloid diseases, including higher relapse rate and inferior survival. The authors suggested that decreased iKIR-HLA C pair should be avoided in ATG based haplo-HSCT settings. In another original article, Zhou et al. identified that patients with CMV and EBV co-reactivation experienced higher incidence of viral pneumonitis, delayed CD4^+^CD25^+^ T cell reconstitution and poor survival. In allo-HSCT settings, Wu et al. highlighted mechanisms underlying increase in EBV viral load, risk factors and treatment for HBsAg-positive donors and recipients, which might allow the inclusion of HBsAg-positive individuals as donors or transplant candidates. Wang and Zhao reviewed the effects of IL-15 on natural killer cell development through activation of several downstream signaling pathways, such as Ras-MEK-MAPK, JAK-STAT5, and PI3K-ATK-mTOR pathways. All of these suggest the advances in factors associated with transplant complications and potential strategies for prevention and treatment of leukemia relapse and virus infection.

This Research Topic “Recent Developments in Haploidentical Stem Cell Transplantation: Therapy and Complications” provides some insights into the recent advances of haplo-HSCT. Moreover, this Research Topic may also contribute to the body of knowledge in haplo-HSCT for the prevention of GF, leukemia relapse, and virus infection as well as the enhancement of the graft-versus-leukemia (GVL) effect. However, challenges remain in the haplo-HSCT settings. For example, could the indications for haplo-HSCT be further expanded? Should pre-HSCT residual disease be eradicated to improve outcomes? Could we identify new subgroup patients who will benefit the strong GVL effect of haplo-HSCT? Could novel strategies for complication prevention or treatment be established through elucidating the underlying mechanisms of hematopoietic recovery and immune reconstitution? etc. Should these challenges be successfully dealt with, *we can teach young dog (haploidentical transplantation) new tricks*.

## Author Contributions

All authors contributed to the article and approved the submitted version.

## Conflict of Interest

The authors declare that the research was conducted in the absence of any commercial or financial relationships that could be construed as a potential conflict of interest.

## Publisher’s Note

All claims expressed in this article are solely those of the authors and do not necessarily represent those of their affiliated organizations, or those of the publisher, the editors and the reviewers. Any product that may be evaluated in this article, or claim that may be made by its manufacturer, is not guaranteed or endorsed by the publisher.

